# E-liquid flavors and nicotine concentration choices over 6 months after a smoking cessation attempt with ENDS: Secondary analyses of a randomized controlled trial

**DOI:** 10.18332/tpc/196136

**Published:** 2025-01-15

**Authors:** Angela Flavia Mosimann, Eva M. Güttinger, Kali Tal, Anna Schoeni, Stéphanie Baggio, Nicolas Sambiagio, Aurélie Berthet, Isabelle Jacot-Sadowski, Jean-Paul Humair, Martin Brutsche, Anja Frei, Moa Lina Haller, Reto Auer, Julian Jakob

**Affiliations:** 1 Institute of Primary Health Care, University of Bern, Bern, Switzerland; 2 Center for Child Health Analytics, Children's Hospital Central Switzerland, Lucerne Cantonal Hospital, Lucerne, Switzerland; 3 Laboratory of Population Health (#PopHealthLab), University of Fribourg, Switzerland; 4 Centre for Primary Care and Public Health (Unisanté), University of Lausanne, Switzerland; 5 Department of Primary Care Medicine, University Hospital of Geneva, Geneva, Switzerland; 6 Lung Center, Kantonsspital St. Gallen, St. Gallen, Switzerland; 7 University of Zurich, Epidemiology, Biostatistics and Prevention Institute, Zurich, Switzerland; 8 Department of Internal Medicine, University Hospital Bern - Inselspital, Bern, Switzerland; 9 Department of Paediatrics, University Hospital Bern - Inselspital, Bern, Switzerland

**Keywords:** e-cigarette, ENDS, e-liquid, flavor, nicotine concentration, smoking cessation

## Abstract

**INTRODUCTION:**

Many tobacco smokers try to quit with electronic nicotine delivery systems (ENDS or e-cigarettes). We aimed to describe e-liquid flavors and nicotine concentration use over 6 months in a prospective cohort of smokers willing to quit with ENDS.

**METHODS:**

We included 622 participants from the intervention group of the Efficacy, Safety and Toxicology of ENDS randomized controlled trial. Participants were adult smokers smoking at least five cigarettes a day. They received free ENDS and a choice of 6 e-liquid flavors in 4 nicotine concentrations and smoking cessation counseling. We tracked flavor choice and nicotine concentration at 1, 2, 4, and 8 weeks, and at 6 months, after the target quit date, comparing participants who reported only vaping (exclusive e-cigarette users) to those who vaped and smoked (dual users) over the last 7 days. We applied multivariable regression models to compute adjusted risk ratios (ARR).

**RESULTS:**

At Week 1, 66% (n=409) were exclusive e-cigarette users, and 21% (n=129) were dual users. At Month 6, 43% (n=266) were exclusive e-cigarette users, and 16% (n=102) were dual users. While flavor choices were similar at Week 1, at 6 months, exclusive e-cigarette users reported using more fruity flavors than dual users (31% vs 22%, ARR=2.10; 95% CI: 1.21–3.66). The nicotine concentration used initially was similar in both groups and diminished over time. At 6 months, exclusive e-cigarette users used a lower mean nicotine concentration than dual users (6.3 vs 8.2 mg/mL, difference= -1.55; 95% CI: -2.84 – -0.25 mg/mL).

**CONCLUSIONS:**

After 6 months, exclusive e-cigarette users used more fruity-flavored e-liquids and chose a lower mean nicotine concentration than dual users.

## INTRODUCTION

E-cigarettes, also called electronic nicotine delivery systems (ENDS) or vapes, are increasingly popular among smokers and are often used to attempt to quit combustible cigarettes^1^. ENDS seems to be an effective and less expensive alternative to nicotine replacement therapy (NRT)^2-4^. Unlike most NRT products, ENDS are available with a broad range of e-liquid flavors and nicotine concentrations, giving smokers a range of choices to potentially substitute cigarettes^5-8^. Smokers who try ENDS have the option to change nicotine concentrations and flavors over time^7,9,10^.

When smokers switch to ENDS, observational data suggests that they tend to initially choose tobacco flavors, but long-term users are likely to report choosing fruity flavors^7,9,10^. The flavor preferences may change over time and users may use different flavors concurrently^10-12^. Flavor variety may even help ex-smokers remain abstinent^9,10^, as some studies reported that limiting flavors may drive them back to smoking cigarettes or dissuade smokers from switching to ENDS^9,13,14^. ENDS users who use e-liquids with nicotine, quit smoking more successfully than those who use e-liquids without nicotine^15-17^.

Studies on the use of ENDS flavors and nicotine concentrations have been mainly cross-sectional, showing prevalence of ENDS use at a single time-point, though it would be preferable to measure changes in vaping or smoking status over time. Since these studies typically included experienced ENDS users, their participants are not likely to represent the population of those who use ENDS to quit smoking. They also rely solely on self-reports, which are often inaccurate measures. The few existing randomized controlled trials of ENDS for smoking cessation restricted participants’ choice of nicotine concentrations and flavors^2,16-18^. The drawback of such RCTs with restricting flavors and nicotine concentration choices is that they do not reflect the typical experience with ENDS use in most countries where many e-liquid flavorings and nicotine concentrations are available. Thus, we need to analyze data from prospective cohorts that follow up smokers from their quit attempt and continue tracking them over time in a context where a choice of flavors and nicotine concentrations are available.

We therefore seized the unique opportunity to use data from the Efficacy, Safety, and Toxicology of ENDS (ESTxENDS) randomized controlled trial^19^, which provided free ENDS devices and 24 combinations of e-liquids to smokers who wanted to quit, along with standard-care smoking cessation counseling. We aimed to describe the choice of e-liquids and nicotine concentrations over time, comparing the use of e-liquid flavors and the liquids’ nicotine concentration between participants who only used ENDS (exclusive e-cigarette users) to those who used ENDS and smoked (dual users), collecting data at 5-time points over a 6-month period.

## METHODS

### Study design, setting, and participants

This is an observational secondary analysis of the ESTxENDS trial, a randomized controlled multicenter trial testing ENDS’ Efficacy, Safety, and Toxicology for smoking cessation (Clinical trial NCT03603340)^19^.

The ESTxENDS trial included 1246 adult smokers, smoking ≥5 cigarettes per day, who were willing to quit smoking with the help of ENDS. Participants were recruited in 5 study sites in Switzerland from July 2018 through June 2021. Participants randomized into the intervention group received free ENDS and a free supply of e-liquids for 6 months (see below), along with standard-care smoking cessation counseling in person and over the phone. Participants from the control group received only smoking cessation counseling. At the baseline visit, the nurses confirmed eligibility and collected written informed consent. We present the data collected from baseline to 6-month follow-up of the 622 participants in the intervention group. We assessed smoking and vaping status, flavor choice, and nicotine concentration during phone calls at 1, 2, 4, and 8 weeks after the target quit date and at an in-person visit at 6 months.

### ENDS device and e-liquids

Participants received two e-cigarette starter kits (Innokin Endura T20-S). At the baseline visit, study nurses instructed the participants how to use ENDS, fill the device with e-liquid, charge the device, and to change the coil every 2 weeks to avoid overheating of the coil.

We offered a choice of 6 e-liquid flavors (menthol, green apple, raspberry, red fruit, and two different tobacco flavors (FR-M and FR4)) in 4 different nicotine concentrations (0, 6, 11, 19.6 mg/mL), all made by Alfaliquid^20^. Participants could sample the 24 e-liquid options from an e-liquid testing board comprising ENDS (one of each combination of flavor and nicotine concentration) and then choose the e-liquid and nicotine concentration they preferred (Supplementary file Figure 1). Participants could use their ENDS *ad libitum* and freely re-order e-liquids in any amount. They could order one type or a mix of flavors, and nicotine concentrations.

### Data sources and measurements

We collected the data via questionnaires. At each consultation, we assessed smoking and vaping status with the following two survey questions: ‘Did you smoke in the last 7 days?’ and ‘Did you use ENDS in the last 7 days?’. We defined participants who reported they had not smoked but had used ENDS in the last 7 days, as exclusive e-cigarette users. We defined participants who reported they had smoked and used ENDS during the previous 7 days, as dual users. We defined participants who reported neither using ENDS nor having smoked in the last 7 days as tobacco and e-cigarette abstainers. We defined participants who reported only smoking in the last 7 days as exclusive smokers. Participants without information on smoking or vaping in the last 7 days, were defined as missing for the concerned visit.

At each follow-up visit, we assessed the flavors and nicotine concentrations that participants had used over the last 7 days. We noted if participants had used e-liquids other than those we provided, and included the flavor and nicotine concentration used in our dataset.

### Flavor choice and nicotine concentration

We classified flavors as tobacco, fruity, menthol, or other. Fruity flavors included green apple, raspberry, red fruit, and other fruity flavors purchased separately by participants. Tobacco included FR-M and FR4 tobacco, as well as other tobacco-flavored e-liquids purchased by participants. We coded flavors that fell outside those groups as ‘other’ (e.g. caramel or cucumber).

We recorded nicotine concentration for all e-liquids reported by participants. If participants used a variety of nicotine concentrations in the same week, we calculated mean nicotine concentration by summing up the nicotine concentrations and dividing them by the number of different concentrations.

### Nicotine exposure in urine

To contrast self-reported nicotine concentration in e-liquids to an objective measure of total nicotine exposure, we calculated creatinine-adjusted total nicotine equivalents (TNE)^21^. To validate self-reported exposures, we quantified anabasine concentrations in urine at baseline and at Month 6 in a subset of participants. Calculation details are given in Supplementary file Methodology.

### Statistical analyses

We used descriptive statistics to determine baseline characteristics. We present the use of flavors (absolute frequencies and percentages) by category (fruity, tobacco, menthol, other flavors, flavor mix) and mean nicotine concentration (mg/mL; mean and standard deviation) over time in figures and tables as the main outcome, separately for exclusive e-cigarette users and dual users. We also present participants (absolute frequencies and percentages) who use e-liquids without nicotine. We compared exclusive e-cigarette users and dual users at each visit (Weeks 1, 2, 4, 8, and Month 6) separately: we applied multivariable logistic regression models to compute adjusted risk ratios (ARR) of use of each e-liquid flavor category separately (tobacco, fruity, menthol, flavor mix or other flavors). We applied multivariable linear regression models to determine nicotine concentration in exclusive e-cigarette users and dual users in each visit separately. Finally, we performed a multivariable logistic regression model to determine if flavor status at Week 1 was associated with smoking status at Month 6 in all participants from the intervention group.

We adjusted all models for confounders such as age, sex, marital status, education level, work situation, age started smoking, cigarettes smoked per day, use of other inhaled tobacco products, secondhand smoke (smoking partner), and the Fagerström test for nicotine dependence (FTND) score (0–10)^22^. The selection of covariables was based on clinical knowledge and previous studies^19,23^. We used inverse probability weighting to account for attrition separately for each visit.

### Analysis of nicotine exposure in urine

To validate self-reported nicotine concentration use, we used multivariable regression models to compare TNE and anabasine levels in exclusive e-cigarette users and dual users at baseline and Month 6. We also applied separate multivariable mixed models to study differences in TNE and anabasine between baseline and Month 6 in exclusive e-cigarette users or dual users. For both models, we used the same covariables as described above.

### Sensitivity analyses: continuous, exclusive e-cigarette users and dual users

We analyzed flavor choice and nicotine concentration in continuous, exclusive e-cigarette users and continuous dual users. We defined continuous, exclusive e-cigarette users as participants who reported they had used only ENDS since their last visit at every visit where data on e-cigarette use and smoking were available. Continuous dual users were participants who reported they smoked and used ENDS since their last visit at every visit where data on e-cigarettes and smoking were available. Time frames between visits varied from one week to six months. Detailed methods of computing continuous, exclusive e-cigarette users and continuous dual users are given in Supplementary file Methodology.

## RESULTS

### Participants

Among the 622 participants of the intervention group of the ESTxENDS study, the median age at baseline was 38 years (IQR: 29–52); 53% identified as men and 47% as women, none identified with another gender identity. The median Fagerström score was 4 (IQR: 3–6). At baseline, participants smoked a median of 15 cigarettes daily (IQR: 10–20) and reported having started smoking at the age of 16 years (IQR: 15–18) (Table 1).

**Table 1:** Sociodemographic characteristics and smoking history among the participants of the intervention group of the ESTxENDS study - descriptive statistics, recruitment 2018–2021 (N=622)

*Characteristics*	*All n (%)*	*Week 1 exclusive cigarette users n (%)*	*Week 1 dual users n (%)*
**Total,** n	622	409	129
**Men**	332 (53)	225 (55)	66 (51)
**Age** (years), median (IQR), range	38 (29–52), 18–79	38 (30–51),19–79	41 (29–56), 18–74
**Education level**			
Obligatory school/other/none	50 (8)	33 (8)	9 (7)
Apprenticeship/high school graduate	291 (47)	186 (45)	67 (52)
Higher degree/university	281 (45)	190 (46)	53 (41)
**Work situation**			
Homemaker/looking for a job/other	127 (20)	71 (17)	39 (30)
Working/studying	495 (80)	338 (83)	90 (70)
**Marital status**			
Single/divorced/widowed/judicially separated	453 (73)	285 (70)	97 (75)
Married/registered partnership	169 (27)	124 (30)	32 (25)
**Smoking history**			
Cigarettes smoked daily at baseline, median (IQR), range	15 (10–20), 5–60	15 (10–20), 5–45	20 (12–20), 6–60
Age when started smoking, median (IQR), range	16 (15–18), 8–46	16 (15–18), 8–32	17 (15–19), 11–46
Smoking other tobacco products in the last 6 months	158 (25)	105 (26)	31 (24)
Secondhand smoke: smoking partner	330 (53)	205 (50)	74 (57)
Fagerström score, median (IQR), range	4 (3–6), 0–10	4 (3–6), 0–10	5 (4–7), 0–10

Percentages may not add up to 100% due to rounding to integer values. IQR: interquartile range.

### Distribution of vaping and smoking status

The number of exclusive e-cigarette users decreased over the visits [66% (n=409) at Week 1 to 43% (n=266) at Month 6]. The number of dual users remained roughly stable over the visits [21% (n=129) at Week 1 to 16% (n=102) in Month 6]. Proportions of tobacco and e-cigarette abstainers and exclusive smokers increased over the visits [at Week 1, 3% (n=18) tobacco and e-cigarette abstainers and 1% (n=5) exclusive smokers; at Month 6, 10% (n=62) tobacco and e-cigarette abstainers and 19% (n=120) exclusive smokers]. The number of missing participants varied between 10% (n=61) and 14% (n=84) at each visit (Supplementary file Figure 2).

### Flavors and nicotine concentration

*Flavor choice*

**Figure 1:**
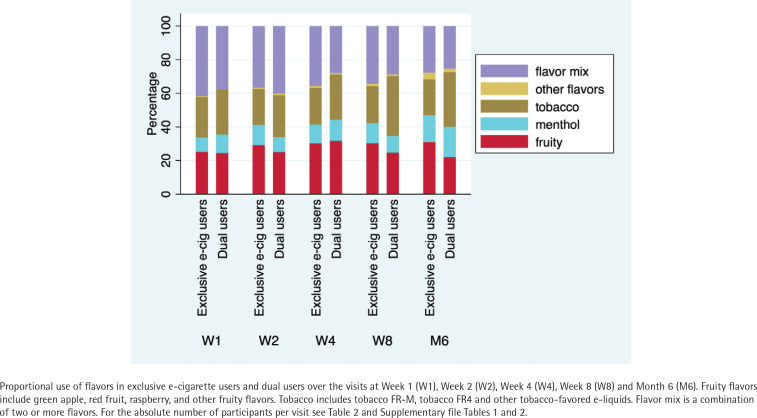
Flavor choice of exclusive e-cigarette users and dual users among the participants of the intervention group of the ESTxENDS study, recruitment 2018–2021 (N=622)

In Week 1, flavor distribution among exclusive e-cigarette users and dual users was: fruity 25% vs 24%; menthol 9% vs 11%; tobacco 24% vs 27%; other flavors were both 0%; and flavor mix 42% vs 38%. Results for Weeks 2, 4, and 8 are given in Supplementary file Table 1. At Month 6, flavor distribution among exclusive e-cigarette users and dual users was: fruity 31% vs 22%, menthol 16% vs 18%, tobacco 21% vs 33%, other flavors 4% vs 2%, and 28% vs 25% mixed their flavors (Figure 1 and Table 2).

**Table 2:** E-liquid flavors and nicotine concentrations at Week 1 and Month 6 among the participants of the intervention group of the ESTxENDS study - descriptive statistics, recruitment 2018–2021

*Variable*	*Week 1*					*Month 6*				
	*Overall n (%)*	*Exclusive e-cigarette users n (%)*	*Dual users n (%)*	*ARR (95% CI)*	*p*	*Overall n (%)*	*Exclusive e-cigarette users n (%)*	*Dual users n (%)*	*ARR (95% CI)*	*p*
**Total,** n	538	409	129			368	266	102		
**Missing**	4 (1)	2 (0.5)	2 (2)			24 (7)	16 (6)	7 (8)		
**Flavors**	534 (100)	407 (100)	127 (100)			344 (100)	249 (100)	95 (100)		
Fruity*	133 (25)	102 (25)	31 (24)	0.92 (0.60–1.42)	0.718	98 (28)	77 (31)	21 (22)	2.10 (1.21–3.66)	0.007
Menthol	49 (9)	35 (9)	14 (11)	0.59 (0.28–1.28)	0.195	57 (17)	40 (16)	17 (18)	0.89 (0.48–1.67)	0.721
Tobacco**	133 (25)	99 (24)	34 (27)	0.86 (0.57–1.29)	0.475	84 (24)	53 (21)	31 (33)	0.49 (0.33–0.71)	0.001
Other flavors***	1 (0)	1 (0)	0 (0)	NA	NA	12 (3)	10 (4)	2 (2)	NA	NA
Flavor mix****	218 (41)	170 (42)	48 (38)	1.29 (0.94–1.77)	0.107	93 (27)	69 (28)	24 (25)	1.14 (0.71–1.84)	0.575
**Flavor combinations**										
Fruity + tobacco	110 (21)	86 (21)	24 (19)			39 (11)	28 (11)	11 (12)		
Fruity + menthol	42 (8)	35 (9)	7 (6)			26 (8)	18 (7)	8 (8)		
Tobacco + menthol	55 (10)	41 (10)	14 (11)			26 (8)	22 (9)	4 (4)		
Any combination with other flavors	3 (0.5)	3 (0.7)	0 (0)			2 (0.6)	1 (0.4)	1 (1)		
>2 combinations	8 (1)	5 (1)	3 (2)			0 (0)	0 (0)	(0)		
Nicotine concentration in e-liquids, n	534	407	127			344	250	94		
**Nicotine concentration** (mg/mL), mean (SD)	12.8 (5.06)	12.5 (5.06)	13.4 (5.01)	-0.41 (-1.62–0.80)	0.508	6.8 (4.60)	6.3 (4.40)	8.2 (4.85)	-1.55 (-2.84 – -0.25)	0.019
Participants who use e-liquids without nicotine	5 (1)	5 (1)	0 (0)			37 (11)	34 (14)	3 (3)		

ARR: adjusted risk ratio. NA: not applicable. Percentages are relative to non-missing observations. Percentages may not add up to 100% due to rounding to integer values.

* Fruity flavors: green apple, raspberry, red fruit, or other fruity flavors (cherry, blood orange, berry, lemon, apricot, peach, nectarine, blueberry, jackfruit, passion fruit, grenadine, mango, and grapes).

** Tobacco flavors: tobacco FR-M, tobacco FR4 or another tobacco flavor.

*** Other flavors: chai, sparkling voddo juic, marchmello, eisenbeer, piña colada, bloody summer, hazelnut, drunken pudding, cucumber, coffee, caramel, and emmentaler drachenfrucht.

**** Flavor mix: a combination of the flavors above.

We found no significant difference in the choice of flavors between exclusive e-cigarette users and dual users during the visits at Weeks 1, 2, and 4. At Week 8 and Month 6, exclusive e-cigarette users reported using significantly fewer tobacco-flavored e-liquids than dual users (Week 8, 22% vs 36%, ARR=0.59; 95% CI: 0.41–0.85, p=0.008; Month 6, 21% vs 33%, ARR=0.49; 95% CI: 0.33–0.71, p<0.001). At Month 6, exclusive e-cigarette users reported using more fruity flavors than dual users (31% vs 22%, ARR=2.10; 95% CI: 1.21–3.66, p=0.007). Proportions of other flavor choices were similar in exclusive e-cigarette users and dual users at Week 8 and Month 6, respectively (Table 2; Supplementary file Tables 1–3 and Figure 3).

One week after receiving their ENDS, 42% of exclusive e-cigarette users and 38% of dual users mixed flavors. At Month 6, 28% of exclusive e-cigarette users and 25% of dual users mixed flavors. In both groups, combining tobacco and fruity-flavored e-liquids was the most popular flavor mix over all visits, followed by tobacco and mint (Table 2; Supplementary file Table 1). Most ENDS users reported using at least one non-tobacco flavored e-liquid over the visits (at Week 1: 76% of exclusive e-cigarette users, 73% of dual users; at Month 6: 79% of exclusive e-cigarette users, 67% of dual users) (Table 2).

*Nicotine concentration in e-liquids*

**Figure 2:**
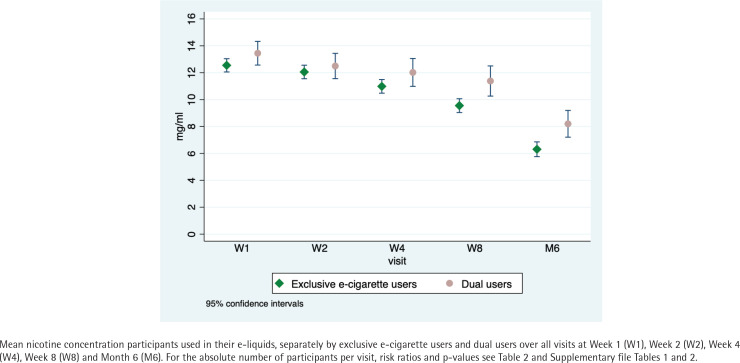
Mean nicotine concentration in e-liquids: exclusive e-cigarette users and dual users among the participants of the intervention group of the ESTxENDS study, recruitment 2018–2021 (N=622)

At Week 1, exclusive e-cigarette users used a mean nicotine concentration of 12.5 mg/mL in their e-liquids vs 13.4 mg/mL used by dual users (coefficient= -0.41; 95% CI: -1.62–0.80, p=0.51). Both groups steadily decreased the mean nicotine concentration over time; differences between exclusive e-cigarette users and dual users at Weeks 1, 2, 4, and 8 did not reach statistical significance (Figure 2; Supplementary file Tables 1 and 2). At Month 6, exclusive e-cigarette users used a lower mean nicotine concentration than dual users (6.3 vs 8.2 mg/mL, difference= -1.55 mg/mL; 95% CI: -2.84 – -0.25, p=0.02). Only a few participants used e-liquids without nicotine (at Week 1: 1 % (n=5) of exclusive e-cigarette users and 0% (n=0) of dual users; at Month 6: 14% (n=34) of exclusive e-cigarette users and 3% (n=3) of dual users) (Table 2). At Month 6, exclusive e-cigarette users who used e-liquids without nicotine used the following flavors: fruity 38% (n=13), tobacco 18% (n=6), menthol 18% (n=6), other flavors 9% (n=3), and flavor mix 18% (n=6). Dual users who used e-liquids without nicotine at Month 6 used the following flavors: fruity 0%, tobacco 33% (n=1), menthol 67% (n=2), other flavors 0%, and flavor mix 0% (Supplementary file Table 6).

### Flavor status and smoking status

Flavor status at Week 1 was not associated with smoking status at Month 6. Odds of still smoking at Month 6 were similar in participants who had used tobacco-flavored e-liquids or fruity-flavored e-liquids at Week 1 (OR=1.12; 95% CI: 0.58–2.18, p=0.734).

### Nicotine exposure in urine

Our sub-sample included urinary TNE measures of 145 participants at baseline [17% dual users at Week 1 (n=24) and 83% exclusive e-cigarette users at Week 1 (n=121)] and 119 participants at Month 6 [27% dual users (n=32) and 73% exclusive e-cigarette users (n=87)]. TNE at Month 6 was lower in exclusive e-cigarette users than in dual users (19.1 vs 25.4 nmol/mg creatinine, coefficient= -8.5; 95% CI: -15.78 – -1.31, p=0.021). Anabasine at Month 6 was also lower in exclusive e-cigarette users than dual users (1.5 vs 4.98 ng/mg creatinine in dual users, coefficient= -4.08; 95% CI: -6.48 – -1.68, p=0.001) (Supplementary file Table 4).

From baseline to Month 6, exclusive e-cigarette users reduced their TNE, whereas dual users increased their TNE: Exclusive e-cigarette users reduced their TNE with a mean difference of -8.17 nmol/mg creatinine (95% CI: -12.18 – -4.15, p<0.001). Dual users increased their TNE with a mean difference of 5.3 nmol/mg creatinine (95% CI: 0.40–10.21, p=0.033). The mean difference of anabasine between baseline and Month 6 in exclusive e-cigarette users was -7.77 ng/mg creatinine (95% CI: -9.93 – -5.60, p<0.001). The mean difference of anabasine between baseline and Month 6 in dual users was -3.28 (95% CI: -6.83–0.28, p=0.071) (Supplementary file Table 5).

### Sensitivity analyses: continuous, exclusive e-cigarette users and dual users

At Week 1, 69% (n= 429) of the users were continuous exclusive e-cigarette users, and 25% (n=157) were continuous dual users. In Month 6, 33% (n=208) of the users were continuous exclusive e-cigarette users, and 8% (n=47) were continuous dual users (Supplementary file Figure 4). At each visit, flavor choices among continuous, exclusive e-cigarette users and continuous dual users were similar to those in exclusive e-cigarette users and dual users with a 7-day prevalence. At Month 6, continuous exclusive e-cigarette users reported using more fruity-flavored ENDS than continuous dual users (32% vs 9%, ARR=3.30; 95% CI: 1.15–9.45, p=0.012). At Week 8, continuous exclusive e-cigarette users reported using less tobacco-flavored ENDS than continuous dual users (21% vs 42%, ARR=0.44; 95% CI: 0.28– 0.70, p=0.002) and at Month 6 (37% vs 20%, ARR=0.28; 95% CI: 0.22–0.65, p=0.003). Other flavor choices were similar in continuous, exclusive e-cigarette users and dual users (Supplementary file Figure 5 and Supplementary file Tables 7 and 8).

Continuous, exclusive e-cigarette users and dual users both decreased their mean nicotine concentration over time (Supplementary file Figure 6). There were no significant differences between the groups at Weeks 1, 2, 4, and 8. At Week 1, continuous, exclusive e-cigarette users used a mean nicotine concentration of 12.5 mg/mL, and continuous dual users used 13.5 mg/mL (p=0.507, difference= -0.40; 95% CI: -1.60–0.76). At Month 6, continuous exclusive e-cigarette users used a significantly lower nicotine concentrations than continuous dual users (5.6 vs 11.0 mg/mL; p=0.004, difference= -3.16 mg/mL; 95% CI: -5.32 – -1.01) (Supplementary file Tables 6 and 7).

## DISCUSSION

Among participants in a smoking cessation trial that offered a choice of free e-liquids over 6 months combined with standard-care smoking cessation counseling, participants’ initial choice of e-liquids varied and remained varied over time. Exclusive e-cigarette users used more fruity-flavored e-liquids at 6 months and fewer tobacco-flavored e-liquids than dual users at 8 weeks and at 6 months. In Weeks 1, 2, and 4, there was no significant difference in flavor choice between exclusive e-cigarette users and dual users. The proportion of participants who used menthol, other, or mixed flavors was similar across groups during all visits. Both groups reduced nicotine concentration over time. At Month 6, exclusive e-cigarette users reported using lower e-liquid nicotine concentrations than dual users, a trend validated by urinary concentrations of nicotine in a sub-sample of participants. E-liquids without nicotine were unpopular. Overall visits, only a few participants (mostly exclusive e-cigarette users) used e-liquids without nicotine. Flavor choice was comparable to what we found in the main results.

Cross-sectional studies^7,9,10^ and a cohort study with one follow-up after 3–5 years^10^, indicated that ENDS users preferred tobacco flavor at initial ENDS use and eventually switched to fruity or other non-tobacco flavors. However, we found that participants initially experimented with different flavors and then stuck with their preferred flavor, continuing its use over time. Most ENDS users reported using at least one non-tobacco-flavored e-liquid over their visits. Exclusive e-cigarette users used more fruity flavors than dual users at 6 months and fewer tobacco flavors at 8 weeks and 6 months after the target quit date. Participants in our study used a wide range of flavors over time. Independent randomized controlled trials comparing restricted choice of flavors are needed to assess the causality of available flavors on smoking cessation outcomes.

The ESTxENDS study found that ENDS were effective in helping people to quit smoking but not to quit nicotine^19^. We found that most ENDS users continued to use e-cigarettes with nicotine-containing e-liquids after they quit smoking. However, exclusive e-cigarette users and dual users reduced e-liquid nicotine concentrations over time. There is evidence that initial low nicotine concentrations may not meet smokers’ needs when switching to e-cigarettes and thus may increase the likelihood that they start smoking again^6^. Higher nicotine levels such as >15 mg/mL may better help smokers quit initially, and nicotine concentration used is likely to drop among tobacco cigarette quitters over time^6^. As in other studies, we found a decrease in self-reported use of nicotine concentrations over time^6,7^. Also, we found a lower total nicotine exposure measured in urine at 6 months after the target quit date in exclusive e-cigarette users. This contradicts the argument that exclusive e-cigarette users could also use their device more frequently with an e-liquid containing less nicotine and thus use lower nicotine concentrations. In contrast, the total nicotine intake would remain the same or even increase. In contrast, dual users showed a higher TNE after 6 months despite the reduction of nicotine concentration in e-liquids. This is probably due to the double exposure to nicotine through e-liquids and tobacco cigarettes. Overall, our study suggests that smokers willing to quit smoking with the help of ENDS may start with e-liquids that contain a high concentration of nicotine and that the people who remain abstinent from smoking can expect to lower the e-liquid concentration and total nicotine intake gradually.

### Limitations

Results and interpretations of the analyses should be considered exploratory. The widths of the confidence intervals for all analyses were not adjusted for multiplicity. The ESTxENDS trial was not designed to test differences in flavor and nicotine concentrations used between groups and was not designed to answer whether the choice of flavor and nicotine concentrations was more effective than a restricted choice of flavors and nicotine concentrations. We urge refraining from the causal interpretation of the study findings on the effectiveness of the choice of flavors and nicotine concentration on tobacco and nicotine abstinence outcomes. Instead, these are *post hoc* exploratory analyses. While we set the level of statistical significance to 5%, it essentially served as a guidance for interpreting these exploratory results. It should not be used in place of hypothesis testing.

Our results rely on self-reported use of e-liquids over time. We validated self-reported exposure to nicotine concentration in urine in a subsample of participants. These validations are limited to the visits at baseline and 6 months.

## CONCLUSIONS

Among participants in a smoking cessation trial that allowed participants to have a broad choice of free e-liquids over 6 months combined with standard-care smoking cessation counseling allowing NRT and further smoking cessation drug therapy, we found that exclusive e-cigarette users used more fruity-flavored ENDS after 6 months and less tobacco-flavored ENDS than dual users at 8 weeks and at 6 months after their target quit date. The proportions of participants reporting using menthol, other, or mixed flavors remained similar across groups over all visits. Exclusive e-cigarette users and dual users reduced their e-liquid nicotine concentration over time, but exclusive e-cigarette users reduced their nicotine concentration even more. Exclusive e-cigarette users reduced their total nicotine equivalent (TNE). In contrast, dual users increased their TNE over time, so smokers quitting with the help of ENDS and wanting to reduce their nicotine intake should be encouraged to switch to ENDS completely and avoid dual use.

## Supplementary Material



## Data Availability

The data supporting this research are available from the authors on reasonable request.
