# Supplementary material for: E-liquid flavors and nicotine concentration choices over 6 months after a smoking cessation attempt with ENDS: Secondary analyses of a randomized controlled trial
Source: Tob Prev Cessat. 2025 Jan 15;11:10.18332/tpc/196136. doi: 10.18332/tpc/196136 (PMC11734314; doi:10.18332/tpc/196136)
Supplement: Supplementary file 1 [file TPC-11-04-s1.pdf]

E-liquid flavors and nicotine concentration choices over 6-months after a smoking cessation attempt with ENDS: secondary analyses of a randomized controlled trial

Appendix: Methodology, Limitations, Figures (6) and Tables (8)

CONTENTS

E-LIQUID FLAVORS AND NICOTINE CONCENTRATION CHOICES OVER 6-MONTHS AFTER A SMOKING CESSATION ATTEMPT WITH ENDS: SECONDARY ANALYSES OF A RANDOMIZED CONTROLLED TRIAL 1

APPENDIX METHODOLOGY 4

ENDS DEVICE AND E-LIQUIDS 4
DATA SOURCES AND MEASUREMENTS 4
NICOTINE EXPOSURE IN URINE 4
STATISTICAL METHODS 5
SENSITIVITY ANALYSIS – *CONTINUOUS* STATUS 5
STATISTICAL ANALYSIS- ANNOTATION 5

APPENDIX LIMITATIONS 5

APPENDIX TABLES 6

APPENDIX TABLE 1) E-LIQUID FLAVORS AND NICOTINE CONCENTRATIONS OF EXCLUSIVE E-CIGARETTE USERS AND DUAL USERS AT ALL VISITS – DESCRIPTIVE STATISTICS 6
APPENDIX TABLE 2) E-LIQUID FLAVORS AND NICOTINE CONCENTRATIONS OF EXCLUSIVE E-CIGARETTE USERS AND DUAL USERS AT ALL VISITS - RISK RATIOS AND P-VALUES 10
APPENDIX TABLE 3) USE OF SPECIFIC E-LIQUID FLAVORS SEPARATELY: EXCLUSIVE E-CIGARETTE USERS AND DUAL USERS ALL VISITS – DESCRIPTIVE STATISTICS 13
APPENDIX TABLE 4) TNE VALUES COMPARING EXCLUSIVE E-CIGARETTE USERS AND DUAL USERS AT BASELINE* AND MONTH 6 15
APPENDIX TABLE 5) TNE VALUES COMPARING NICOTINE USE AT BASELINE* TO MONTH 6 IN EXCLUSIVE E-CIGARETTE USERS AND DUAL USERS – COEFFICIENTS AND P-VALUES 16
APPENDIX TABLE 6) PARTICIPANTS CHARACTERISTICS AND FLAVOR CHOICE OF EXCLUSIVE E-CIGARETTE USERS AND DUAL USERS WHO USED E-LIQUIDS WITHOUT NICOTINE AT MONTH 6 – DESCRIPTIVE STATISTICS 18
APPENDIX TABLE 7) E-LIQUID FLAVORS AND NICOTINE CONCENTRATIONS OF *CONTINUOUS* EXCLUSIVE E-CIGARETTE USERS AND DUAL USERS AT ALL VISITS – DESCRIPTIVE STATISTICS 20

Appendix table 8) E-liquid flavors and nicotine concentrations comparing *continuous* exclusive e-cigarette users and dual users at all visits - adjusted risk ratios and p-values 22

APPENDIX FIGURES 25

Appendix figure 1) Photograph of the wooden board presenting the ENDS filled with e-liquids and flavors available 25
Appendix Figure 2) Distribution of vaping and smoking status over the visits 25
Appendix Figure 3) Minigraphs of flavor choice of exclusive e-cigarette users and dual users 27
Appendix Figure 4) Distribution of continuous exclusive e-cigarette users and continuous dual users over all visits 28
Appendix Figure 5) Flavor choice of *continuous* exclusive e-cigarette users and *continuous* dual users over all visits 29
Appendix Figure 6) Mean nicotine concentration in *continuous* exclusive e-cigarette users *continuous* and dual users over all visits 30

Appendix Methodology

ENDS device and e-liquids

Participants received two e-cigarette starter kits (Innokin Endura T20-S). The Endura T20-S kit, produced in China by Innokin, came in a user packet with a 1,500 mAh internal Li-Po battery, a Prism S Coil (0.8 Ohm, 16-18W) atomizer, a spare drip tip, a micro-USB DC 5V/1A cable and an instruction manual in French or German. To avoid financial conflicts of interest with the manufacturer, the study team paid Innokin its factory price for the kits and spare coils and paid to ship the e-liquids to our study centers. Study participants were not charged for the materials we provided. At the Baseline visit, study nurses told the participants how to use ENDS, fill the device with e-liquid, charge the device, and change the coil every 2 weeks to avoid overheating of the coil.

We offered a choice of 6 e-liquid flavors (menthol, green apple, raspberry, red fruit and two different tobacco flavors (FR-M and FR4)) in 4 different nicotine concentrations (0, 6, 11, 19.6 mg/ml), all made by *Alfaliquid.*[19] Participants could sample the 24 e-liquid options from an e-liquid testing board comprising ENDS (one of each combination of flavor and nicotine concentration) and then pick the e-liquid and nicotine concentration they preferred (Appendix Figure 1). Study nurses provided participants with a maximum of 10 e-liquid bottles (10 ml per bottle) at the end of their Baseline visit and advised them to use only the liquids we provided. Participants could order new e-liquids during scheduled phone visits or contact the study nurses during office hours to re-order e-liquids or coils, or replace faulty devices. Participants could use their ENDS ad libitum and freely re-order e-liquids in any amount. They could order one type or a mix of flavors or nicotine concentrations.

Data sources and measurements

Nicotine exposure in Urine

To contrast self-reported nicotine concentration in e-liquids to an objective measure of total nicotine exposure, we calculated creatinine-adjusted Total Nicotine Equivalents (TNE)[20], which corresponds to the molar sum of nicotine, cotinine, 3-OH-cotinine and norcotinine adjusted by the concentration of urinary creatinine. TNE was calculated from the four metabolites in urine at Baseline and Month 6 separately using the following calculation: TNE= ((nicotine/162.23)+(cotinine/176.22)+(3-OH-cotinine/192.21)+(norcotinine/162.19)).

To validate self-reported exposures, we quantified anabasine concentrations in urine, at Baseline, and at Month 6 in a subset of participants. Nicotine, cotinine,

OH-cotinine, norcotinine and anabasine were quantified in urine using a liquid chromatography–mass spectrometry (LC-MS) method. Analyses were performed routinely by University Center of Legal Medicine (Lausanne, Switzerland).

Statistical methods

Sensitivity analysis – *continuous* status

As described in the manuscript, we defined continuous exclusive e-cigarette users as participants who reported they had used only ENDS since their last visit at every visit where data on e-cigarette use and smoking was available.

Example 1: A participant was an exclusive e-cigarette user at Week 1 and was also an exclusive e-cigarette user at Week 2, then this person counts as continuous exclusive e-cigarette user at the Week 2 visit. The same would be for a person that was not available for visit at Week 1, but states at Week 2 that he/she exclusively used e-cigarettes since the last visit (which would then be the baseline visit).

Example 2: A participant was an exclusive e-cigarette user at Week 1 and 2, but no data was available for Week 4. At the 6 Month visit, the participant claims to have used only e-cigarettes since the last visit (in his/her case the last visit was at Week 2), then this participant also counts as continuous e-cigarette user at the Month 6 visit.

The same applies analogously for dual users. This is why time frames between visits can vary from one Week to six Months.

Statistical analysis- annotation

The widths of the confidence intervals for all analyses were not adjusted for multiplicity and should be interpreted as exploratory. The ESTxENDS trial was not designed to test differences in flavor and nicotine concentrations used between groups. These are post-hoc exploratory analyses. While we set the level of statistical significance to 5%, it served essentially as a guidance for interpretation of these exploratory results.

Appendix Limitations

We designed the ESTxENDS intervention with a range of e-liquids flavor and nicotine concentrations to simulate the wide range of flavors and nicotine concentration in the e-liquid market in Switzerland and in most countries where these are available. We did not provide guidance to participants on which flavor

they should use but let them choose. Also, while we encouraged participants to start with high nicotine concentrations at Baseline, we did not set goals to have them reduce nicotine concentrations over time or set a nicotine-abstinence date. We let them order as many e-liquids they wanted, whenever they wanted and of any nicotine concentration they wanted. This enabled us to observe what would happen in a clinical setting when no specific guidance over time is provided. Future studies should test the effect of tailored counseling over time for the choice of e-liquids.

ESTxENDS has scheduled follow-ups at 12-, 24-, and 60- months which will provide information on the behavior of participants beyond 6-months.

Appendix Tables

Appendix Table 1) E-liquid flavors and nicotine concentrations of exclusive e-cigarette users and dual users at all visits – descriptive statistics

**Variable**	**Week 1**			**Week 2**		**Week 4**		**Week 8**		**Month 6**		
	**Overall**	**Exclusive e-cigarette users**	**Dual users**	**Exclusive e-cigarette users**	**Dual users**	**Exclusive e-cigarette users**	**Dual users**	**Exclusive e-cigarette users**	**Dual users**	**Overall**	**ENDS only users**	**Dual users**
**N total**<br>N missing (%)	**538**<br>4 (1)	**409**<br>2 (0.5)	**129**<br>2 (2)	**403**<br>2 (0.5)	**113**<br>1 (1)	**390**<br>2 (0.5)	**105**<br>1 (1)	**353**<br>3 (1)	**102**<br>1 (1)	**368**<br>24 (7)	**266**<br>16 (6)	**102**<br>7 (8)
**Flavors N (%)**	**534 (100)**	**407 (100)**	**127 (100)**	**401 (100)**	**112 (100)**	**388 (100)**	**104 (100)**	**350 (100)**	**101 (100)**	**344 (100)**	**249 (100)**	**95 (100)**
Fruity*	133 (25)	102 (25)	31 (24)	117 (29)	28 (25)	117 (30)	33 (32)	106 (30)	25 (25)	98 (28)	77 (31)	21 (22)

Menthol	49 (9)	35 (9)	14 (11)	48 (12)	10 (9)	43 (11)	13 (13)	42 (12)	10 (10)	57 (17)	40 (16)	17 (18)
Tobacco**	133 (25)	99 (24)	34 (27)	86 (21)	28 (25)	85 (22)	28 (27)	77 (22)	36 (36)	84 (24)	53 (21)	31 (33)
Other flavors***	1 (0)	1 (0)	0 (0)	3 (1)	1 (1)	5 (1)	1 (1)	5 (1)	1 (1)	12 (3)	10 (4)	2 (2)
Flavor mix****	218 (41)	170 (42)	48 (38)	147 (37)	45 (40)	138 (36)	29 (28)	120 (34)	29 (29)	93 (27)	69 (28)	24 (25)
**Thereof:**												
Fruity + Tobacco	110 (21)	86 (21)	24 (19)	64 (16)	19 (17)	61 (16)	9 (9)	49 (14)	14 (14)	39 (11)	28 (11)	11 (12)
Fruity + menthol	42 (8)	35 (9)	7 (6)	35 (9)	9 (8)	36 (9)	7 (7)	32 (9)	4 (4)	26 (8)	18 (7)	8 (8)
Tobacco + menthol	55 (10)	41 (10)	14 (11)	45 (11)	17 (15)	37 (10)	11 (11)	34 (10)	9 (9)	26 (8)	22 (9)	4 (4)
Fruity + other	2 (0.4)	2 (0.5)	0 (0)	0 (0)	0 (0)	0 (0)	2 (2)	1 (0.3)	1 (1)	1 (0.3)	0 (0)	1 (1)
Tobacco + other	1 (0.2)	1 (0.25)	0 (0)	0 (0)	0 (0)	0 (0)	0 (0)	0 (0)	0 (0)	1 (0.3)	1 (0.4)	0 (0)
Menthol + other	0 (0)	0 (0)	0 (0)	0 (0)	0 (0)	0 (0)	0 (0)	0 (0)	0 (0)	0 (0)	0 (0)	0 (0)
Fruity + tobacco + menthol	6 (1)	4 (1)	2 (2)	3 (0.75)	0 (0)	4 (1)	0 (0)	4 (1)	0 (0)	0 (0)	0 (0)	0 (0)

Fruity+ menthol+ other	0 (0)	0 (0)	0 (0)	0 (0)	0 (0)	0 (0)	0 (0)	0 (0)	1 (1)	0 (0)	0 (0)	0 (0)
Tobacco + menthol + other	0 (0)	0 (0)	0 (0)	0 (0)	0 (0)	0 (0)	0 (0)	0 (0)	0 (0)	0 (0)	0 (0)	0 (0)
Fruity + tobacco + other	2 (0.4)	1 (0.25)	1 (0.8)	0 (0)	0 (0)	0 (0)	0 (0)	0 (0)	0 (0)	0 (0)	0 (0)	0 (0)
**Nicotine concentration N**	**534**	**407**	**127**	**401**	**112**	**388**	**104**	**350**	**100**	**344**	**250**	**94**
Mean nicotine concentration (mg/ml) [SD]	12.8 [5.06]	12.5 [5.06]	13.4 [5.01]	12.1 [5.13]	12.5 [5.02]	11.0 [5.08]	12.0 [5.33]	9.6 [4.89]	11.4 [5.65]	6.8 [4.60]	6.3 [4.40]	8.2 [4.85]

Comments: Percentages are relative to non-missing observations. Since rounded to whole numbers, the percentage may not add up to 100 %.

* Fruity flavors: green apple, raspberry, red fruit, or other fruity flavors (cherry, blood orange, berry, lemon, apricot, peach, nectarine, blueberry, jackfruit, passion fruit, grenadine, mango, and grapes)

** Tobacco flavors: tobacco FR-M, tobacco FR4 or another tobacco flavor

***Other flavors: chai, sparkling voddo juic, marchmello, eisenbeer, piña colada, bloody summer, hazelnut, drunken pudding, cucumber, coffee, caramel, and emmentaler drachenfrucht.

**** Flavor mix: a combination of the flavors above

<u>Abbreviations</u>: N = number, SD = standard deviation

Appendix table 2) E-liquid flavors and nicotine concentrations of exclusive e-cigarette users and dual users at all visits - risk ratios and p-values

Risk ratio and p-value for using a flavor vs. not using it, comparing exclusive e-cigarette users and dual users at each visit.

**Variable**	**Week 1**		**Week 2**		**Week 4**		**Week 8**		**Month 6**	
**Flavor**	**ARR (95% CI)**	**p-value**	**ARR (95% CI)**	**p-value**	**ARR (95% CI)**	**p-value**	**ARR (95% CI)**	**p-value**	**ARR (95% CI)**	**p-value**
**Fruity**										
unadjusted	0.96 (0.62, 1.47)	0.841	1.01 (0.68, 1.49)	0.959	0.96 (0.64, 1.42)	0.829	1.34 (0.84, 2.14)	0.210	1.67 (0.95, 2.95)	0.061
multivariable-adjusted	0.92 (0.60, 1.42)	0.718	0.97 (0.67, 1.40)	0.861	0.85 (0.58, 1.24)	0.417	1.31 (0.82, 2.10)	0.250	2.10 (1.21, 3.66)	0.007
**Menthol**										
unadjusted	0.74 (0.34, 1.61)	0.447	1.71 (0.67, 4.33)	0.253	0.88 (0.43, 1.79)	0.721	0.97 (0.46, 2.06)	0.940	0.92 (0.49, 1.73)	0.800
multivariable-adjusted	0.59 (0.28, 1.28)	0.195	1.55 (0.61, 3.98)	0.349	0.89 (0.41, 1.94)	0.779	1.23 (0.59, 2.57)	0.583	0.89 (0.48, 1.67)	0.721
**Tobacco**										
unadjusted	0.96 (0.64, 1.44)	0.846	0.95 (0.62, 1.47)	0.820	0.88 (0.57, 1.37)	0.573	0.61 (0.42, 0.88)	0.012	0.55 (0.37, 0.83)	0.005

multivariable-adjusted	0.86 (0.57, 1.29)	0.475	0.89 (0.58, 1.37)	0.608	0.90 (0.57, 1.43)	0.669	0.59 (0.41, 0.85)	0.008	0.49 (0.33, 0.71)	0.001
Other aromas										
	Too few participants	Too few participants	Too few participants	Too few participants	Too few participants	Too few participants	Too few participants	Too few participants	Too few participants	To few participants
**Flavor mix**										
unadjusted	1.12 (0.82, 1.52)	0.472	0.93 (0.68, 1.24)	0.588	1.17 (0.81, 1.70)	0.387	1.31 (0.87, 1.96)	0.180	1.23 (0.76, 1.98)	0.390
multivariable-adjusted	1.29 (0.94, 1.77)	0.107	1.00 (0.73, 1.35)	0.984	1.25 (0.86, 1.80)	0.225	1.21 (0.83, 1.87)	0.264	1.14 (0.71, 1.84)	0.575
**Nicotine concentration**	**Coefficient (95% CI)**	**p-value**	**Coefficient (95%CI)**	**p-value**	**Coefficient (95%CI)**	**p-value**	**Coefficient (95%CI)**	**p-value**	**Coefficient (95%CI)**	**p-value**
unadjusted	-1.10 (-2.36, 0.15)	0.083	-0.14 (-1.33, 1.10)	0.822	-0.93 (-2.27, 0.42)	0.176	-1.53 (-2.96, -0.11)	0.035	-1.99 (-3.42, -0.57)	0.006
multivariable-adjusted	-0.41 (-1.62, 0.80)	0.508	0.40 (-0.70, 1.51)	0.475	-0.65 (-1.92, 0.63)	0.318	-1.02 (-2.30, 0.26)	0.118	-1.55 (-2.84, -0.25)	0.019

Comments: We compared exclusive e-cigarette users and dual users at each visit (Week 1, 2, 4, 8, and Month 6) separately: We applied multivariable-adjusted logistic regression models to compute adjusted risk ratios (ARR) of use of each e-liquid flavor category separately (tobacco, fruity, menthol, flavor mix or other flavors). We applied multivariable-adjusted linear regression models to determine nicotine concentration in exclusive e-cigarette users and dual users in each visit separately.

We adjusted all models for demographics (age, sex, education level, work situation, marital status) and smoking history (No. of cigarettes smoked daily at baseline, age when started smoking, smoking other tobacco products in the last 6 months, secondhand smoke (smoking partner), Fagerstrom score). For all models we used inverse probability weighting to account for attrition.

Abbreviations: ARR = adjusted risk ratio, 95% CI = 95% confidence interval

Appendix table 3) Use of specific e-liquid flavors separately: Exclusive e-cigarette users and dual users all visits – descriptive statistics

Number of participants using a specific flavor. Fitting with Appendix Figure 3).

**Variable**	**Week 1**			**Week 2**		**Week 4**		**Week 8**		**Month 6**		
	**Overall**	**Exclusive e-cigarette users**	**Dual users**	**Exclusive e-cigarette users**	**Dual users**	**Exclusive e-cigarette users**	**Dual users**	**Exclusive e-cigarette users**	**Dual users**	**Overall**	**Exclusive e-cigarette users**	**Dual users**
**N total (%)**	**538 (100)**	**409 (100)**	**129 (100)**	**403 (100)**	**113 (100)**	**390 (100)**	**105 (100)**	**353 (100)**	**102 (100)**	**368 (100)**	**266 (100)**	**102 (100)**
Number n and percentage (%) of participants who used the following flavors. Several flavors could be used at the same time.												
**Fruity***	**295 (55)**	**230 (56)**	**65 (50)**	**219 (54)**	**56 (50)**	**218 (56)**	**51 (49)**	**192 (54)**	**45 (54)**	**164 (45)**	**123 (46)**	**41 (40)**
Green apple	111 (21)	92 (23)	19 (15)	79 (20)	16 (14)	78 (20)	19 (18)	70 (20)	13 (13)	56 (18)	37 (14)	19 (19)
Raspberry	98 (18)	76 (19)	22 (17)	77 (19)	21 (19)	79 (20)	18 (17)	72 (20)	19 (19)	48 (13)	34 (13)	14 (14)
Red fruit	135 (25)	103 (25)	32 (25)	106 (26)	26 (23)	112 (29)	27 (26)	94 (27)	22 (22)	88 (24)	72 (27)	16 (16)
Other fruity flavors***	3 (0.56)	3 (0.73)	0 (0)	4 (1)	1 (1)	1 (0)	0 (0)	5 (1)	1 (1)	9 (2)	8 (3)	1 (1)

**Menthol**	**152 (28)**	**155 (28)**	**37 (29)**	**131 (33)**	**36 (32)**	**120 (31)**	**31 (30)**	**112 (32)**	**24 (24)**	**109 (30)**	**80 (30)**	**29 (28)**
**Tobacco****	**307 (57)**	**232 (57)**	**75 (58)**	**198 (49)**	**64 (57)**	**187 (48)**	**48 (46)**	**164 (47)**	**59 (58)**	**150 (41)**	**104 (39)**	**46 (45)**
FR4 tobacco	158 (29)	118 (29)	40 (31)	107 (27)	33 (29)	105 (27)	23 (22)	88 (25)	30 (29)	70 (19)	51 (19)	19 (19)
FR-M tobacco	171 (32)	128 (31)	43 (33)	107 (27)	35 (31)	100 (26)	30 (28)	83 (24)	37 (36)	81 (22)	51 (19)	30 (29)
Other tobacco flavors	2 (0.85)	1 (0.56)	1 (2)	1 (0.49)	1 (2)	0 (0)	1 (2)	3 (2)	0 (0)	7 (3)	6 (4)	1 (2)
**Other flavors******	**6 (1)**	**5 (1)**	**1 (1)**	**3 (1)**	**1 (1)**	**5 (3)**	**3 (3)**	**6 (2)**	**3 (3)**	**14 (4)**	**11 (4)**	**3 (3)**

Comments: *Number and participants who used one or more fruity flavors (green apple, raspberry, red fruit, or other fruity flavors)

**Number of participants who used tobacco flavors (FR-M, FR4 or another tobacco flavor)

***Other fruity flavors: cherry, blood orange, berry, lemon, apricot, peach, nectarine, blueberry, jackfruit, passion fruit, grenadine, mango, and grapes

****Other flavors: chai, sparkling voddo juic, marchmello, eisenbeer, piña colada, bloody summer, hazelnut, drunken pudding, cucumber, coffee, caramel, and emmentaler drachenfrucht.

Abbreviations: N = number

Appendix Table 4) TNE values comparing exclusive e-cigarette users and dual users at Baseline* and Month 6

Variable	Baseline						Month 6					
	**Exclusive e-cigarette users at Week 1**	**Dual users at Week 1**	**Unadjusted Coefficient (95% CI)**	**Unadjusted p-value**	**Adjusted coefficient (95% CI)**	**Adjusted p-value**	**Exclusive e-cigarette users**	**Dual users**	**Unadjusted coefficient (95% CI)**	**Unadjusted p-value**	**Adjusted coefficient (95% CI)**	**Adjusted p-value**
N total	121	24					87	32				
TNE in nmol/mg creatinine (SD)	28.8 (4.2)	30.0 (6.8)	-1.17 (-9.27, 6.92)	0.775	0.09 (-8.46, 8.64)	0.984	19.1 (4.5)	25.4 (6.5)	-6.35 (-14.31, 1.59)	0.116	-8.5 (-15.78, -1.31)	0.021
Anabasine (ng/mg creatinine)	10.1 (2.6)	10.1 (2.2)	0.06	0.974	0.11 (-3.40, 3.62)	0.949	1.50 (0.50)	4.98 (2.14)	-3.47 (-5.69, -1.25)	0.002	-4.08 (-6.48, -1.68)	0.001

Comments:

TNE is the molar sum of nicotine, cotinine, 3-OH-cotinine and norcotinine adjusted by the concentration of urinary creatinine, calculated at Baseline and Month 6 separately:

TNE= ((nicotine/162.23)+(cotinine/176.22)+(3-OH-cotinine/192.21)+(norcotinine/162.19))

We used multivariable-adjusted regression models to compare exclusive e-cigarette users and dual users at Week 1 and Month 6 separately. We adjusted for age, sex, marital status, education status, work situation, age of starting smoking, cigarette per day, inhalation of other tobacco products, second-hand smoke (smoking partner), and the Fagerstrom score. We used inverse probability weighting to account for attrition separately for Week 1 and Month 6.

Abbreviations: N = Number, 95% CI = 95% confidence interval, SD = standard deviation

*Urine measures took place at Baseline, the status "exclusive e-cigarette user" or "dual user" was defined at Week 1.

Appendix Table 5) TNE values comparing nicotine use at Baseline* to Month 6 in exclusive e-cigarette users and dual users – coefficients and p-values

Variable	Exclusive e-cigarette users				Dual users			
N total	203				56			
	**Unadjusted coefficient (95% CI)**	**Unadjusted p-value**	**Adjusted coefficient**	**Adjusted p-value**	**Unadjusted coefficient (95% CI)**	**Unadjusted p-value**	**Adjusted coefficient**	**Adjusted p-value**
TNE in nmol/mg creatinine	-8.07 (-12.09, -4.04)	< 0.001	-8.17 (-12.18, -4.15)	< 0.001	5.3 (0.40, 10.21)	0.034	5.3 (0.42, 10.23)	0.033
Anabasine (ng/mg creatinine)	-7.78 (-9.91, -5.58)	< 0.001	-7.77 (-9.93, -5.60)	< 0.001	-3.35 (-6.84, 0.13)	0.059	-3.28 (-6.83, 0.28)	0.071

Comments:

TNE is the molar sum of nicotine, cotinine, 3-OH-cotinine and norcotinine adjusted by the concentration of urinary creatinine, calculated at Baseline and Month 6 separately:

TNE= ((nicotine/162.23)+(cotinine/176.22)+(3-OH-cotinine/192.21)+(norcotinine/162.19))

Multivariable-adjusted mixed models to compare differences in TNE between Baseline and Month 6 and used separate models for exclusive e-cigarette users and dual users. We adjusted for age, sex, marital status, education status, work situation, age of starting smoking, cigarette per day, inhalation of other tobacco products, second-hand smoke (smoking partner), and the Fagerstrom score. We used inverse probability weighting to account for attrition separately for exclusive e-cigarette users and dual users.

Abbreviations: N = Number, 95% CI = 95% confidence interval

*Urine measures took place at Baseline, the status “exclusive e-cigarette user” or “dual user” was defined at Week 1.

Appendix Table 6) Participants characteristics and flavor choice of exclusive e-cigarette users and dual users who used e-liquids without nicotine at Month 6 – descriptive statistics

**Characteristics**	Unit	All participants who used 0 mg/ml nicotine at Month 6	Exclusive e-cigarette users at Month 6	Dual users at Month 6
**Participants n**	N	37	34	3
**Men**	N (%)	13 (35)	12 (35)	1 (33)
**Age**	Median (p25; p75)	37.0 (28.0; 49.5)	37.5 (29.5; 50.0)	28.0 (25.0; 37.0)
**Education**	No. (%)			
Obligatory school / other / none		2 (5)	2 (6)	0 (0)
Apprenticeship / high school graduate		20 (54)	18 (53)	2 (67)
Higher degree / University		15 (41)	14 (41)	1 (33)
**Work situation**	**No. (%)**			
Homemaker / Looking for a job / other		7 (19)	7 (21)	0 (0)
Working / studying		30 (81)	27 (79)	3 (100)
**Marital status**	**No. (%)**			
Single / Divorced / Widowed / judicially separated		30 (81)	27 (79)	3 (100)
Married / registered partnership		7 (19)	7 (21)	0 (0)

**Smoking history**				
No. of cigarettes smoked daily at baseline	Median (p25, p75)	15.0 (10.0; 20.0)	15.0 (10.0; 20.0)	15.0 (15.0; 15.0)
Age when started smoking	Median (p25, p75)	17.0 (15.0; 19.0)	16.0 (15.0; 19.0)	18.0 (17.0; 24.0)
Smoking other tobacco products in the last 6 months	No. (%)	5 (14)	4 (12)	1 (33)
Secondhand smoke: smoking partner	No. (%)	27 (73)	24 (71)	3 (100)
Fagerstrom score	Median (p25, p75)	4.0 (2.5; 6.0)	4.0 (2.0; 6.0)	3.0 (3.0; 5.0)
**Flavors at Month 6**	N (%)			
Fruity*		13 (35)	13 (38)	0 (0)
Menthol		7 (19)	6 (18)	1 (33)
Tobacco**		8 (22)	6 (18)	2 (67)
Other flavors***		3 (8)	3 (9)	0 (0)
Flavor mix****		6 (16)	6 (18)	0 (0)

Comments: Percentages are relative to non-missing observations. Since rounded to whole numbers, the percentage may not add up to 100 %.

* Fruity flavors: green apple, raspberry, red fruit, or other fruity flavors (cherry, blood orange, berry, lemon, apricot, peach, nectarine, blueberry, jackfruit, passion fruit, grenadine, mango, and grapes)

** Tobacco flavors: tobacco FR-M, tobacco FR4 or another tobacco flavor

***Other flavors: chai, sparkling voddo juic, marchmello, eisenbeer, piña colada, bloody summer, hazelnut, drunken pudding, cucumber, coffee, caramel, and emmentaler drachenfrucht.

**** Flavor mix: a combination of the flavors above.

Appendix Table 7) E-liquid flavors and nicotine concentrations of *continuous* exclusive e-cigarette users and dual users at all visits – descriptive statistics

**Variable**	**Week 1**			**Week 2**		**Week 4**		**Week 8**		**Month 6**		
	**Overall**	**Cont. Exclusive e-cigarette users**	**Cont. Dual users**	**Cont. Exclusive e-cigarette users**	**Cont. Dual users**	**Cont. Exclusive e-cigarette users**	**Cont. Dual users**	**Cont. Exclusive e-cigarette users**	**Cont. Dual users**	**Cont. Overall**	**Cont. ENDS only users**	**Cont. Dual users**
**N total**<br>N missing (%)	**586**<br>52 (9)	**429**<br>23 (5)	**157**<br>29 (18)	**391**<br>22 (6)	**111**<br>25 (23)	**356**<br>19 (68)	**88**<br>19 (22)	**302**<br>20 (7)	**66**<br>14 (21)	**253**<br>32 (13)	**206**<br>19 (9)	**47**<br>12 (26)
**Flavors**<br>**N (%)**	**534 (100)**	**406 (100)**	**128 (100)**	**369 (100)**	**86 (100)**	**337 (100)**	**69 (100**	**282 (100)**	**52 (100)**	**221 (100)**	**186 (100)**	**35 (100)**
Fruity*	133 (25)	101 (25)	32 (25)	109 (30)	20 (23)	100 (30)	17 (25)	88 (31)	12 (23)	62 (28)	59 (32)	3 (9)
Menthol	49 (9)	35 (7)	14 (11)	46 (12)	9 (10)	42 (12)	8 (12)	38 (14)	7 (13)	35 (16)	30 (16)	5 (14)

Tobacco**	133 (25)	99 (24)	34 (27)	79 (21)	21 (24)	74 (22)	19 (28)	59 (21)	22 (42)	51 (23)	38 (20)	13 (37)
Other flavors***	1 (0)	1 (0.25)	0 (0)	2 (0.54)	0 (0)	4 (1)	0 (0)	5 (2)	0 (0)	9 (4)	8 (4)	1 (3)
Flavor mix****	218 (41)	170 (42)	48 (38)	133 (36)	36 (42)	117 (35)	25 (36)	92 (33)	11 (21)	64 (29)	51 (27)	13 (37)
**Nicotine concentration N**	**534**	**406**	**128**	**369**	**86**	**337**	**69**	**282**	**52**	**221**	**187**	**34**
Mean nicotine concentration (mg/ml) [SD]	12.8 [5.06]	12.5 [5.07]	13.5 [5.00]	11.9 [5.11]	13.0 [4.92]	10.8 [5.00]	11.9 [5.25]	9.0 [4.73]	12.4 [5.52]	6.5 [4.90]	5.6 [4.38]	11.0 [5.17]

Comments: Percentages are relative to non-missing observations. Since rounded to whole numbers, the percentage may not add up to 100 %.

* Fruity flavors: green apple, raspberry, red fruit, or other fruity flavors (cherry, blood orange, berry, lemon, apricot, peach, nectarine, blueberry, jackfruit, passion fruit, grenadine, mango, and grapes)

** Tobacco flavors: tobacco FR-M, tobacco FR4 or another tobacco flavor

***Other flavors: chai, sparkling voddo juic, marchmello, eisenbeer, piña colada, bloody summer, hazelnut, drunken pudding, cucumber, coffee, caramel, and emmentaler drachenfrucht.

**** Flavor mix: a combination of the flavors above

Abbreviations: N = number, SD = standard deviation, cont. = continuous

Appendix table 8) E-liquid flavors and nicotine concentrations comparing *continuous* exclusive e-cigarette users and dual users at all visits - adjusted risk ratios and p-values

**Variable**	**Week 1**		**Week 2**		**Week 4**		**Week 8**		**Month 6**	
**Flavor**	**ARR (95% CI)**	**p-value**	**ARR (95% CI)**	**p-value**	**ARR (95% CI)**	**p-value**	**ARR (95% CI)**	**p-value**	**ARR (95% CI)**	**p-value**
**Fruity**										
unadjusted	0.92 (0.60, 1.40)	0.687	1.68 (0.67, 1.73)	0.750	1.29 (0.74, 2.23)	0.353	1.47 (0.76, 2.86)	0.233	3.69 (1.19, 11.42)	0.013
multivariable-adjusted	0.89 (0.59, 1.35)	0.599	1.04 (0.66, 1.62)	0.866	1.17 (1.17 CI 0.69)	0.549	1.43 (0.73, 2.80)	0.271	3.30 (1.15, 9.45)	0.012
**Menthol**										
unadjusted	0.75 (0.34, 1.64)	0.469	1.29 (0.51, 3.26)	0.582	0.89 (0.47, 1.95)	0.772	0.73 (0.32, 1.69)	0.470	1.2 (0.44, 3.28)	0.719
multivariable-adjusted	0.60 (0.28, 1.29)	0.201	1.13 (0.44, 2.92)	0.793	0.84 (0.37, 1.91)	0.681	0.88 (0.33, 2.36)	0.791	1.66 (0.55, 4.93)	0.362
**Tobacco**										
unadjusted	0.98 (0.65, 1.46)	0.902	1.01 (0.61, 1.70)	0.958	0.91 (0.54, 1.55)	0.737	0.49 (0.31, 0.78)	0.006	0.46 (0.27, 0.81)	0.014

multivariable-adjusted	0.88 (0.58, 1.31)	0.528	0.93 (0.56, 2.55)	0.790	0.89 (0.52, 1.54)	0.689	0.44 (0.28, 0.70)	0.002	0.28 (0.22, 0.65)	0.003
Other flavors										
	Too few participants	Too few participants	Too few participants	Too few participants	Too few participants	Too few participants	Too few participants	Too few participants	Too few participants	Too few participants
**Flavor mix**										
unadjusted	1.13 (0.83, 1.54)	0.415	0.87 (0.62, 1.21)	0.415	0.89 (0.62, 1.30)	0.564	1.72 (0.89, 3.35)	0.086	0.83 (0.46, 1.51)	0.551
multivariable-adjusted	1.30 (0.95, 1.79)	0.092	0.96 (0.68, 1.36)	0.817	0.97 (0.67, 1.43)	0.894	1.83 (0.94, 3.56)	0.057	0.86 (0.45, 1.63)	0.649
**Nicotine concentration**	**Coefficient (95% CI)**	**p-value**	**Coefficient (95% CI)**	**p-value**	**Coefficient (95% CI)**	**p-value**	**Coefficient (95% CI)**	**p-value**	**Coefficient (95% CI)**	**p-value**
unadjusted	-1.14 (-2.38, 0.10)	0.072	-0.73 (-2.10, 0.64)	0.297	-0.57 (-2.12, 0.98)	0.473	-2.46 (-4.50, -0.42)	0.018	-4.52 (-6.81, -2.23)	0.000
multivariable-adjusted	-0.40 (-1.60, 0.79)	0.507	0.02 (-1.21, 1.25)	0.974	0.15 (-1.30, 1.61)	0.836	-0.95 (-2.75, 0.85)	0.299	-3.16 (-5.32, -1.01)	0.004

Με σχόλια [A1]: I changed aroma to flavor. Make sure we use the same term all over the manuscript (flavor)

Comments: We compared *continuous* exclusive e-cigarette users and dual users at each visit (Week 1, 2, 4, 8, and Month 6) separately: We applied multivariable-adjusted logistic regression models to compute adjusted risk ratios (ARR) of use of each e-liquid flavor category separately (tobacco,

fruity, menthol, flavor mix or other flavors). We applied multivariable-adjusted linear regression models to determine nicotine concentration in continuous exclusive e-cigarette users and dual users in each visit separately.

We adjusted all models for demographics (age, sex, education level, work situation, marital status) and smoking history (No. of cigarettes smoked daily at baseline, age when started smoking, smoking other tobacco products in the last 6 months, secondhand smoke (smoking partner), Fagerstrom score). For all models we used inverse probability weighting to account for attrition.

Abbreviations: ARR = adjusted risk ratio, 95% CI = 95% confidence interval

Appendix Figures

Appendix figure 1) Photograph of the wooden board presenting the ENDS filled with e-liquids and flavors available

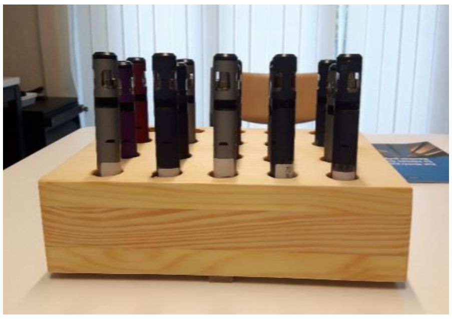

We designed a wooden cardboard for each study site pre-filled with each of the 24 available e-liquid combinations (6 flavors in 4 different nicotine concentrations) available for selection by participants. Participants could test as many as they wanted and as often as they wanted during the baseline visit.

Appendix Figure 2) Distribution of vaping and smoking status over the visits

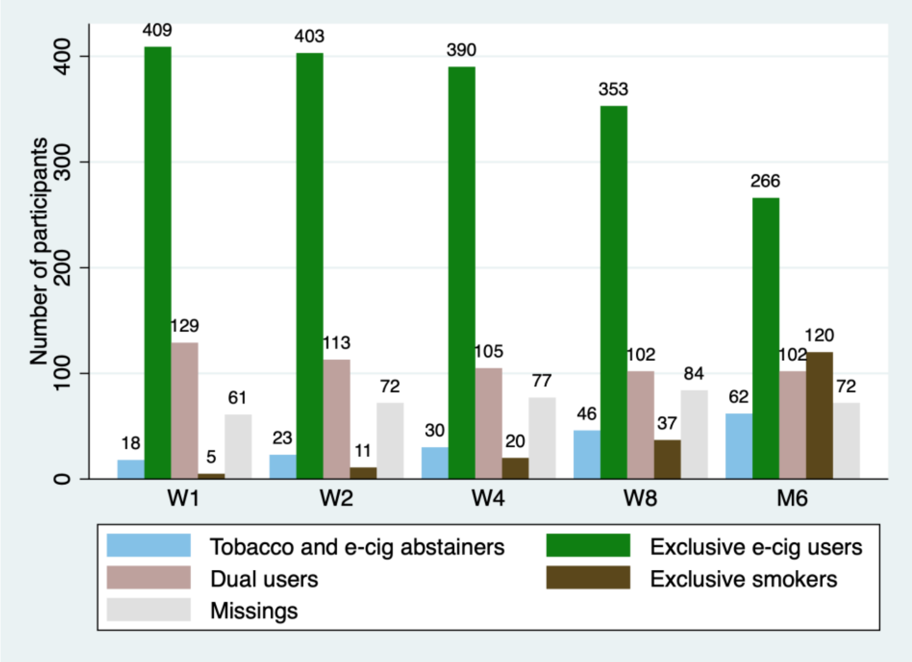

Numerical distribution of all the 622 participants from the intervention group at the visits at Week 1 (W1), Week 2 (W2), Week 4 (W4), Week 8 (W8), and Month 6 (M6). We classified into the following groups: tobacco and e-cigarette abstainers (participants who did not smoke and did not use ENDS during the last 7 days), exclusive e-cigarette users (participants who have not smoked but used ENDS during the last 7 days), dual users (participants who have smoked and used ENDS during the last 7 days), and exclusive smokers (participants who have smoked but not used ENDS during the last 7 days). Participants without information on smoking or vaping status were defined as missing for the concerned visit.

Appendix Figure 3) Minigraphs of flavor choice of exclusive e-cigarette users and dual users

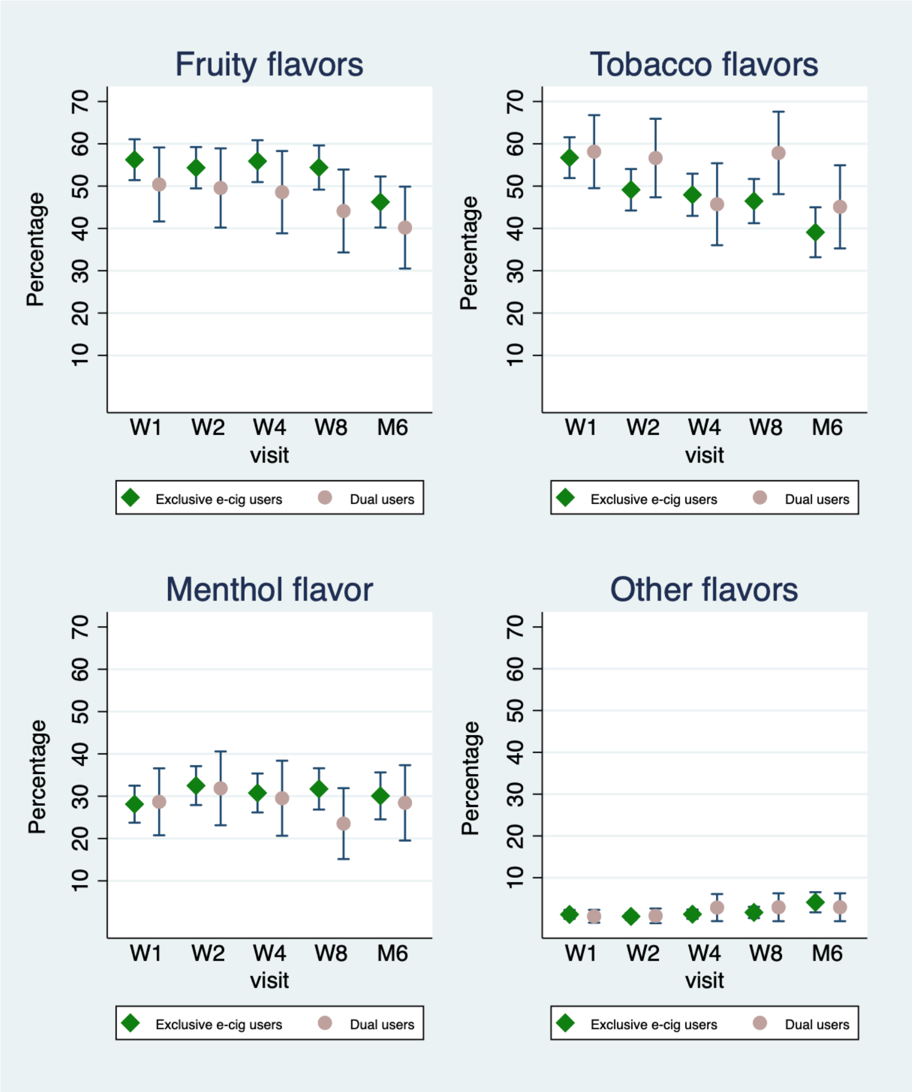

The figures show the percentage of exclusive e-cigarette users and dual users who used fruity flavors (green apple, redfruit, raspberry or other fruity flavors), tobacco flavor (FR-M, FR4, or another tobacco flavor), menthol or other flavors. Participants could use several flavors at a time. Participants who mixed flavors are included in several mini-graphs.

Appendix Figure 4) Distribution of continuous exclusive e-cigarette users and continuous dual users over all visits

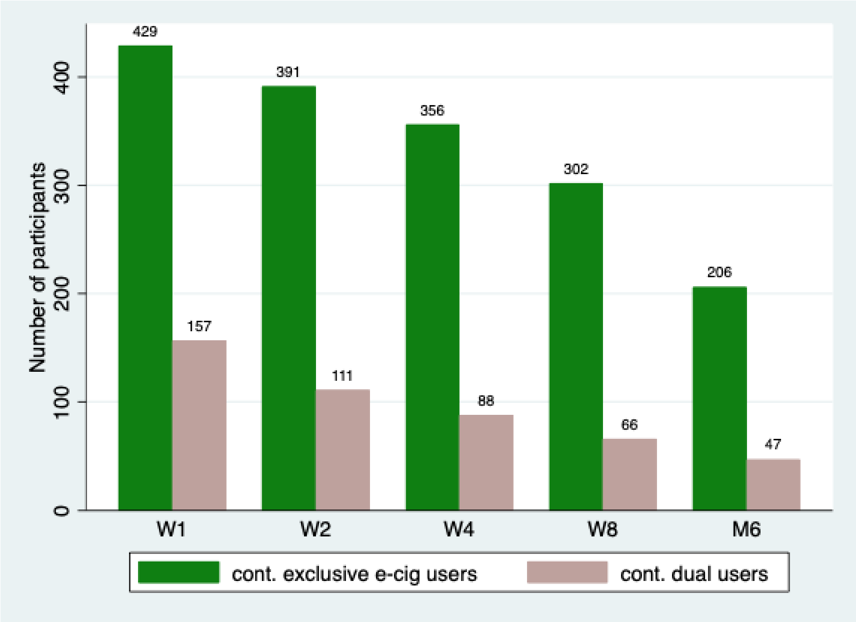

Number of participants who used ENDS and had not smoked since their last visit where data on e-cigarettes use and smoking was available (continuous exclusive e-cigarette users) and participants who used ENDS and had smoked since their last visit where data on e-cigarette use and smoking was available (continuous dual users). Visits were at the target quit date and at Week 1 (W1), Week 2 (W2), Week 4 (W4), Week 8 (W8) and Month 6 (M6). For example, *continuous* exclusive e-cigarette users shown at Week 1 have used only ENDS since their target quit date. *Continuous* exclusive e-cigarette users shown at Week 2 have used only ENDS since Week 1 and so on. Time frames between visits varied from one week to six months.

Appendix Figure 5) Flavor choice of *continuous* exclusive e-cigarette users and *continuous* dual users over all visits

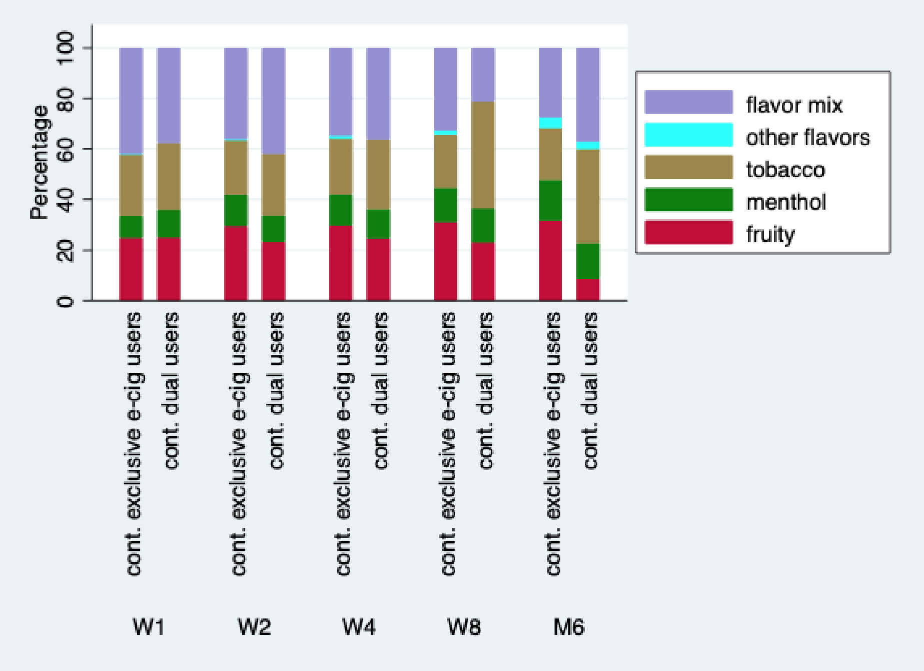

Proportional use of flavors in continuous exclusive e-cigarette users and continuous dual users over the visits at Week 1 (W1), Week 2 (W2), Week 4 (W4), Week 8 (W8) and Month 6 (M6). Fruity flavors include green apple, red fruit, raspberry, and other fruity flavors. Tobacco includes tobacco FR-M, tobacco FR4 and other tobacco flavors. Flavor mix is a combination of two or more flavors.

Appendix Figure 6) Mean nicotine concentration in *continuous* exclusive e-cigarette users *continuous* and dual users over all visits

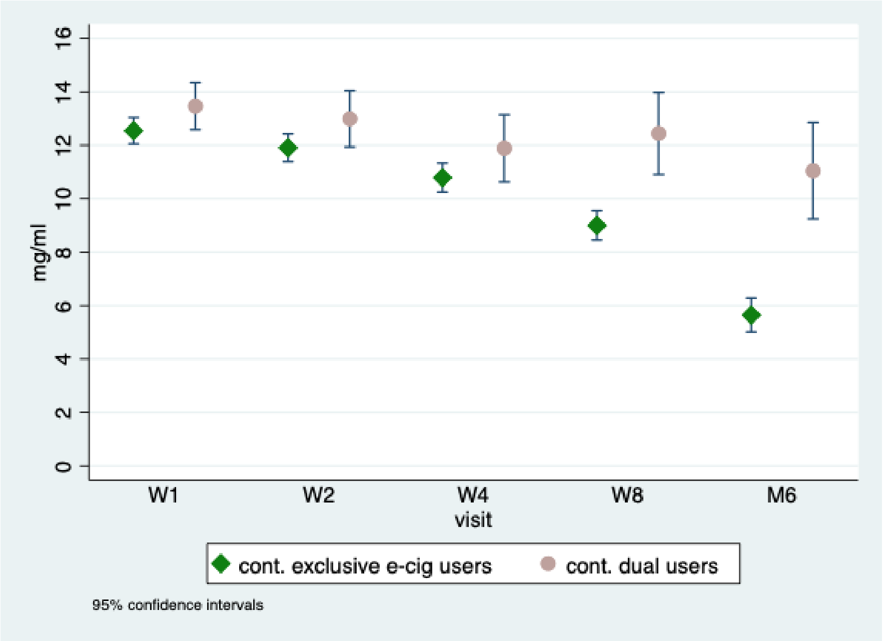

Mean nicotine concentration participants used with ENDS, distinguished between *continuous* exclusive e-cigarette users and *continuous* dual users.

© 2025 Mosimann A.F. et al.
